# Genetic descriptor search algorithm for predicting hydrogen adsorption free energy of 2D material

**DOI:** 10.1038/s41598-023-39696-0

**Published:** 2023-08-05

**Authors:** Jaehwan Lee, Seokwon Shin, Jaeho Lee, Young-Kyu Han, Woojin Lee, Youngdoo Son

**Affiliations:** 1 Department of Industrial and Systems Engineering, Dongguk University-Seoul, Seoul, 04620 South Korea; 2 Data Science Laboratory (DSLAB), Dongguk University-Seoul, Seoul, 04620 South Korea; 3 Department of Energy and Materials Engineering, Dongguk University-Seoul, Seoul, 04620 South Korea; 4 School of AI Convergence, Dongguk University-Seoul, Seoul, 04620 South Korea

**Keywords:** Computer science, Information technology, Cheminformatics

## Abstract

Transition metal dichalcogenides (TMDs) have emerged as a promising alternative to noble metals in the field of electrocatalysts for the hydrogen evolution reaction. However, previous attempts using machine learning to predict TMD properties, such as catalytic activity, have been shown to have limitations in their dependence on large amounts of training data and massive computations. Herein, we propose a genetic descriptor search that efficiently identifies a set of descriptors through a genetic algorithm, without requiring intensive calculations. We conducted both quantitative and qualitative experiments on a total of 70 TMDs to predict hydrogen adsorption free energy ($\Delta G_H$) with the generated descriptors. The results demonstrate that the proposed method significantly outperformed the feature extraction methods that are currently widely used in machine learning applications.

## Introduction

The discovery of graphene^1^ has drawn significant interest to the study of the chemical properties of two-dimensional (2D) materials. Among 2D materials, transition metal dichalcogenides (TMDs) have attracted significant attention due to their unique electronic and optical properties, thus making them promising candidates for various applications in the field of nanoelectronics, optoelectronics, and energy storage. These properties make TMDs suitable for various applications, such as catalysis, energy storage, and sensing. As a result, many recent studies^2–4^ in materials science have focused on the synthesis, characterization, and applications of TMDs.

In the electrocatalytic hydrogen evolution reaction (HER), the hydrogen adsorption free energy on the surface of TMDs substantially determines their catalytic performance^5,6^. This parameter reflects the strength of the interaction between the TMD surface and hydrogen atoms during the HER process. The optimal hydrogen adsorption free energy value for a catalyst should be close to thermo-neutral, meaning that the catalyst should bind hydrogen with neither too strong nor too weak a force. The optimal hydrogen adsorption free energy value ensures that the catalyst can effectively facilitate hydrogen-related reactions with optimal catalytic activity^7,8^. Therefore, it is crucial to have a thorough understanding of the hydrogen adsorption free energy on TMD surfaces for optimizing their catalytic performance in HER.

The quantum mechanical model is often used to predict the chemical properties, such as the hydrogen adsorption free energy, of materials. This model predicts properties by simulating the surface density of charges within the atoms with respect to their potential functions. The most widely adopted model among the quantum mechanics-based methods is the density functional theory (DFT)^9,10^, which calculates the electron density and electronic structure through wave functions. While DFT calculations has been applied to study HER in various systems, this approach remains computationally expensive^10–15^.

Various deep learning-based approaches have been used to predict chemical properties to address computational challenge. Advances in deep learning algorithms and methods for representing the structure of chemical molecules, such as the simplified molecular-input line-entry system (SMILES)^16–18^ and molecular graph^19–21^, have led to significant performance improvements in chemical property prediction. However, deep neural network-based approaches require extensive training datasets to avoid overfitting, and they may not generalize well without sufficient training samples.

Another approach, descriptor search, aims to identify new descriptors that can represent chemical properties by combining the known primary features of chemicals (e.g., number of electrons, period, atomic weight). In Ran et al.^22^, 5 of the 27 fundamental chemical properties were selected by applying Pearson correlation screening and gradient boosting to data composed of 70 TMD materials to explore the property most related to the hydrogen adsorption free energy of the TMD material. Subsequently, 5 selected properties and 12 prototypical functions were used to construct the 954 combinations as candidates. Then, LEF, LEs, and Vtmx were selected as the optimal descriptors combination which are the most suitable combination for hydrogen adsorption free energy prediction using linear regression (see Table in SI^22^). Recently, sure-independence screening and sparsifying operator (SISSO)^23^, an effective descriptor search method, has been introduced and successfully applied to various material science tasks^5,24,25^. One of SISSO’s strengths lies in its optional capability to leverage domain expertise and prior knowledge in the descriptor selection process. By utilizing their understanding of underlying principles and properties of the domain, users can guide the algorithm to focus on relevant primary features, leading to potential improvements in predictive performance and interpretability of the results. However, when using the SISSO, it is essential to consider the computational efficiency due to the potentially huge search space for descriptor selection.

Therefore, in this study, we propose an efficient and effective descriptor search algorithm called Genetic Descriptor Search (GDS), which overcomes the computational limitations of SISSO by efficiently exploring the feature space through symbolic regression based on genetic algorithms to find optimal descriptors. To validate the efficacy of the proposed algorithm, we conduct experiments on the prediction of hydrogen adsorption free energy of TMDs. The results demonstrate that GDS outperforms various feature selection methods commonly used in machine learning and SISSO. Additionally, we perform a qualitative evaluation using t-SNE^26^ visualization on the descriptor set obtained through the proposed algorithm.

The main contributions of this study are as follows.

- We propose a novel descriptor search method, GDS, which does not need to explore all possible immense feature space, making the descriptor search for TMDs’ hydrogen adsorption free energy more computationally efficient.
- GDS outperforms other feature selection and descriptor search algorithms in predicting $\Delta G_H$ of 70 TMD materials on the quatitative evaluations.
- The qualitative analysis through visualization verifies that the descriptors obtained by GDS can represent the intrinsic relationship between $\Delta G_H$ and TMD.
- Finally, GDS finds the reasonable descriptors that are matched to the relevant literature without using domain expertise.

## Preliminaries

In this section, we describe fundamental parts of the proposed method. First, we explain the symbolic regression utilized by our proposed algorithm for descriptor search, and we then describe the genetic algorithm used to increase the efficiency of the symbolic regression.

### Symbolic regression

Symbolic regression analysis^27^ is a method for finding a mathematical expression that accurately models a dependent variable. The expressions are initially generated through a random combination of mathematical components such as operators, constants, and independent variables. This approach avoids the existence of human bias in the modeling process and can therefore be implemented without prior knowledge of the domain. It also enables the identification of intrinsic relationships between the independent and dependent variables, thus allowing the model to capture the underlying relationships presented in the dataset.

In contrast to traditional regression analysis, which finds the optimal parameters based on a predetermined model structure with fixed independent variables, symbolic regression is more flexible and adaptable approach that directly obtains the optimal model structure and parameters from the data. This increases the search space complexity, potentially leading to an infinite number of possible solutions, which highlights the need for the implementation of appropriate constraints or algorithms to ensure efficiency.

**Figure 1:**
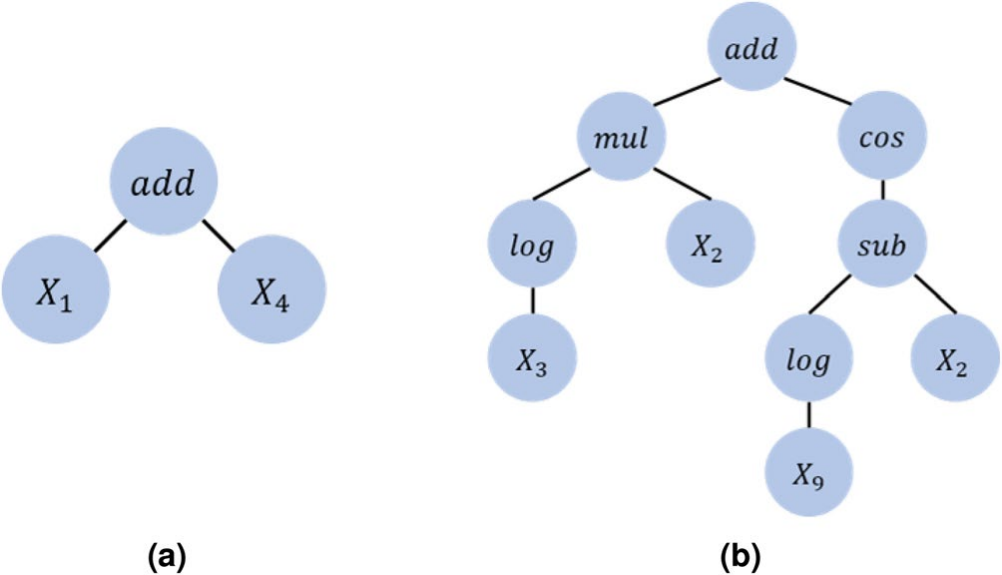
Examples of expression tree with (**a**) a single operator and (**b**) six operators including the root node.

An example of symbolic regression analysis is depicted in Fig. 1. The mathematical function can be represented through an expression tree, which consists of a binary tree of operators and operands. The operators are represented as branch nodes, while the operands are represented as unconditional leaf nodes. In Fig. 1a, the expression tree represents the formula $X_1+X_4$, similarly, in Fig. 1b, the tree represents the formula $X_2{log X_3} + cos(log X_9 - X_2)$. Symbolic regression analysis ultimately aims to select the expression, composed of independent variables, that provides the best representation of the dependent variable.

### Genetic algorithm

Genetic algorithm (GA)^28^ is a population-based meta-heuristic optimization technique that emulates the process of natural evolution. Inspired by Darwin’s theory of evolution, GA operates on the principle of survival of the fittest, where the fittest individuals have higher chances of reproducing and passing their beneficial genes to the next generation.

GA has been successfully applied in a wide range of fields, including engineering, humanities, natural sciences, and even video games^29–33^. The algorithm has proven to be an effective method for solving complex optimization problems in which, the solution space is vast and so traditional optimization techniques may not work effectively.

The core idea behind GA is to encode potential solutions as a set of chromosomes and use genetic operators, such as crossover and mutation, to generate offspring that inherit traits from their parents. The fitness function is used to evaluate the chromosomes and determine their suitability for the problem at hand. Over multiple generations, the population of chromosomes evolves, as the fittest individuals are selected to produce offspring and pass their genes to the next generation. The goal of GA is to find the global optimization by gradually refining the population through this process.

The GA process can be divided into four stages: initial population generation, fitness evaluation, parent selection, and offspring generation through genetic operators. The present study uses a complexity-penalized coefficient of determination as the fitness function and tournament selection^29^ as the parent selection method. This ensures that the GA process remains diverse and well-balanced, which leads to a higher probability of finding the global optimization. The implementation of the GA process of the proposed method is described in detail in the next chapter.

## Proposed method

In this section, we introduce our proposed algorithm, GDS, which is designed to obtain a descriptor set, $\mathfrak {D}$, for predicting chemical properties such as the hydrogen adsorption free energy of TMDs. The main goal of GDS is to generate a set of descriptors that have a high correlation with the chemical property being predicted, which would improve the accuracy of the predictions. To achieve this goal, GDS repeats a three-step descriptor exploration process, which we describe in this section. The iteration is repeated until GDS obtains the desired number of *M* descriptors.

This section begins by discussing the fitness score that we defined, which is a key component of our algorithm. We then provide a detailed description of each step that make up the overall process for obtaining the descriptor that best fits the target residual. The overall procedure to obtain the descriptor set is summarized in Algorithm 1.

### Fitness score

The fitness score for a genetic algorithm is a metric used to evaluate the effectiveness of a specific solution for a given task. In this study, we use a tree as a candidate solution for the descriptor set, and the task is to utilize this set to predict the hydrogen adsorption free energy. In GDS, the descriptors are represented as an expression tree with a maximum depth of lambda, which is obtained by symbolic regression. Increasing the depth of the tree can improve its relationship with the hydrogen adsorption free energy. However, there is a trade-off between depth and complexity. The tree becomes more complex as the depth increases, which negatively impacts the algorithm’s computational cost and interpretability.

To evaluate the impacts of both depth and the relationship with the hydrogen adsorption free energy, we defined the fitness scores of each tree, $p_{i}$, for the *m*-th iteration of the descriptor exploration as follows:

1 $$\begin{aligned}{} & {} \text {score}\;s_i=R^2\left( \Delta _{\mathfrak {D}_{m}}, p_{i}\right) -\lambda \times depth^i \end{aligned}$$

2 $$\begin{aligned}{} & {} \Delta _{\mathfrak {D}_{m}}={\textbf {y}} - \hat{{\textbf {y}}}_{m-1} \end{aligned}$$

3 $$\begin{aligned}{} & {} \hat{{\textbf {y}}}_{0}= {\textbf {0}} \end{aligned}$$

4 $$\begin{aligned}{} & {} \hat{{\textbf {y}}}_{m}={\left( {\mathfrak {D}_{m}}^T \mathfrak {D}_{m}+\alpha I\right) }^{-1} {\mathfrak {D}_{m}}^T {\textbf {y}} \end{aligned}$$

where ${\textbf {y}}$ is the target property and $\Delta _{\mathfrak {D}_{m}}$ is the target residual calculated using ($m-1$)-descriptors set, $\mathfrak {D}_{m-1}$.

In Eq. (1), the first term is the coefficient of determination, which is commonly used in regression analysis to measure the fitness between the dependent and independent variables^34^. Notably, GDS measures the fitness between each tree and the target residual obtained using Eq. (2) with given $m - 1$ descriptors, rather than the fitness between the hydrogen adsorption free energy and each tree. The second term regularizes the complexity of the expression tree by penalizing the fitness with the depth with the control parameter $\lambda$ to prevent the bloat phenomenon, which means the evolution keeps increasing the size of trees without a significant increase in fitness score.

### Initialization of population

In the first step of descriptor exploration, GDS creates the population $P_1$ with given primary features of TMDs, which are represented as $X \in \mathbb {R}^{N \times p}$, where *N* and *p* denote the numbers of the TMD materials and their primary features, respectively. The initial population is obtained by randomly generating $N_{pop}$ trees from the combination of primary features and operator set *H*, defined as

5 $$\begin{aligned} H\equiv \left\{ I,+,-,\times ,\div ,^2 ,^{-1},\sqrt{,}\,log,sin,cos,tan,exp,| |\right\} . \end{aligned}$$

Specifically, the initialization of the population is equal to creating $N_{pop}$ expression trees in Fig. 1. The leaf nodes of trees are randomly selected from the primary features, and other nodes are selected from the operator set *H* in Eq. (5).

### Evolution using tournament selection

In the evolution step, GDS iteratively evolves the initialized population using the fitness score and genetic algorithm. We use the tournament selection strategy^29^ for the genetic algorithm to guarantee diversity in the population, and there is an increased possibility of premature convergence to sub-optimal solution.

The tournament selection is a useful and robust selecting strategy that randomly selects $\tau$-trees from the current population to form a sub-group and run a tournament among them. The winner of the tournament is a candidate with the highest fitness score and it becomes a parent tree that leaves a child for the next generation. In this way, weaker candidates have a chance of being selected, as they do not need to compete with stronger ones unless they are in the same sub-group. To attain a consecutive population, the sub-group needs to be randomly selected $N_{pop}$ times, which reveals the importance of choosing appropriate $N_{pop}$ to reduce the computation burden.

**Figure 2:**
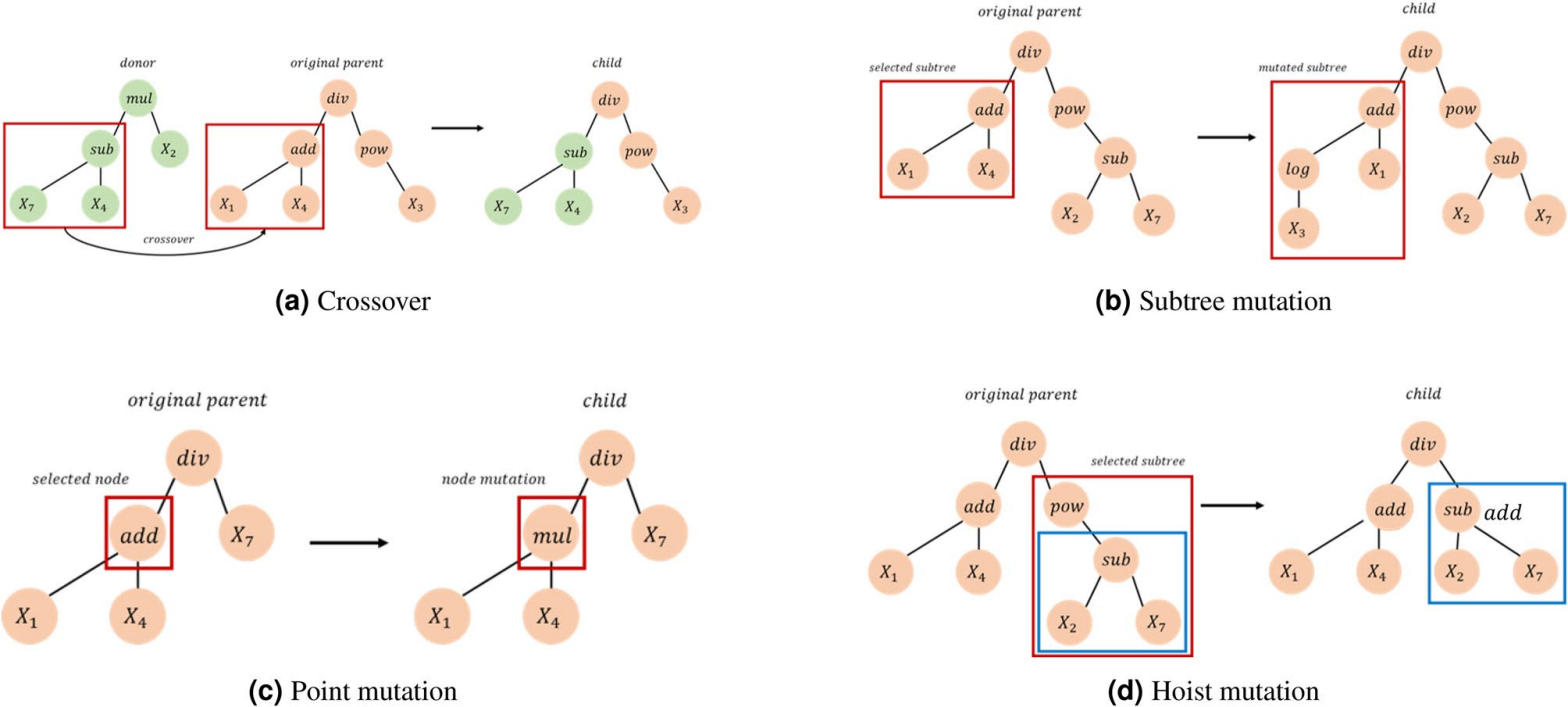
Illustrations of genetic operators: (**a**) crossover needs of two parents, while (**b**–**d**) can be applied to a single parent.

Specifically, we apply the tournament selection strategy to select parent trees with the highest fitness scores from the current population. To generate offspring, the selected parent trees undergo genetic variation, which is achieved by applying genetic operators chosen from the set $\Gamma$. In our study, $\Gamma$ includes reproduction, crossover, and three different mutation methods, which are detailed in Fig. 2.

Reproduction simply clones the parent tree and adds it to the next population unchanged, thus preserving the characteristics of the original population. Crossover (Fig. 2a) combines two parent trees by randomly selecting subtrees from each and swapping them to create a new offspring. Subtree mutation (Fig. 2b) selects a random subtree from the parent trees and replaces it with a new one that has been randomly generated. Point mutation (Fig. 2c) selects a random node from the parent tree and replaces it with one of the operators specified in (5). Lastly, hoist mutation (2d) involves selecting a random subtree and one of its own subtrees. The original subtree is then replaced by this second subtree, which reduces the complexity in the tree and mitigates the bloat phenomenon.

### Update of descriptor set

Once GDS reaches one of the stopping criteria, such as the maximum number of iterations, a tree in the final population with the highest fitness score is chosen as the descriptor, *d*. In our implementation, we use the pre-defined number of generations as the stopping criterion. Then, GDS adds the descriptor *d* to the descriptor set $\mathfrak {D}$ and updates the target residual, $\Delta _{\mathfrak {D}_{m}}$ for the next iteration. Finally, GDS obtains a set of *M* desired descriptors by repeating the three-step descriptor exploration.
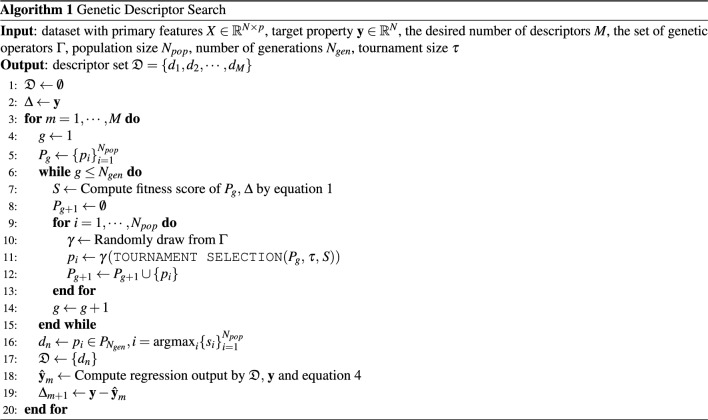


## Results

The performance of the proposed descriptor search algorithm in predicting the hydrogen adsorption free energy ($\Delta G_H$) of TMDs was evaluated using a dataset of 70 TMDs and their corresponding chemical properties. The evaluation was conducted using both quantitative and qualitative approaches. For the quantitative evaluation, the performance of the prediction model using the descriptors from GDS was compared to other relevant methods. Meanwhile, the qualitative analysis involved visualizing the descriptor distribution through the t-SNE^26^, as well as examining the meaning of the chemical properties utilized in the generated descriptors.

### Dataset description

In this study, data was collected for 70 TMDs, as was done in a previous study^22^. In total, 27 features were selected as primary features, as outlined in Table SI3^22^. The process of data collection is detailed below:

15 features (Rtm, Etm, Wtm, Qtm, Vtm, VEtm, VFtm, Vx, Qx, Wx, WFx, Rx, DVEx, EIx, and EItm) out of the 27 primary features were obtained from publicly available databases^35,36^. Another eight features (Ntm, Ntmf, Nx, Nxs, Nxf, Cx, and Covh), which were related to the number of transition metal or chalcogenide atoms, were extracted from the structure utilized in prior study^22^. The remaining five features (Ctm, LEs, LEf, Vtmx, and BEtmx) were derived through calculation.

### Quantitative evaluation

**Figure 3:**
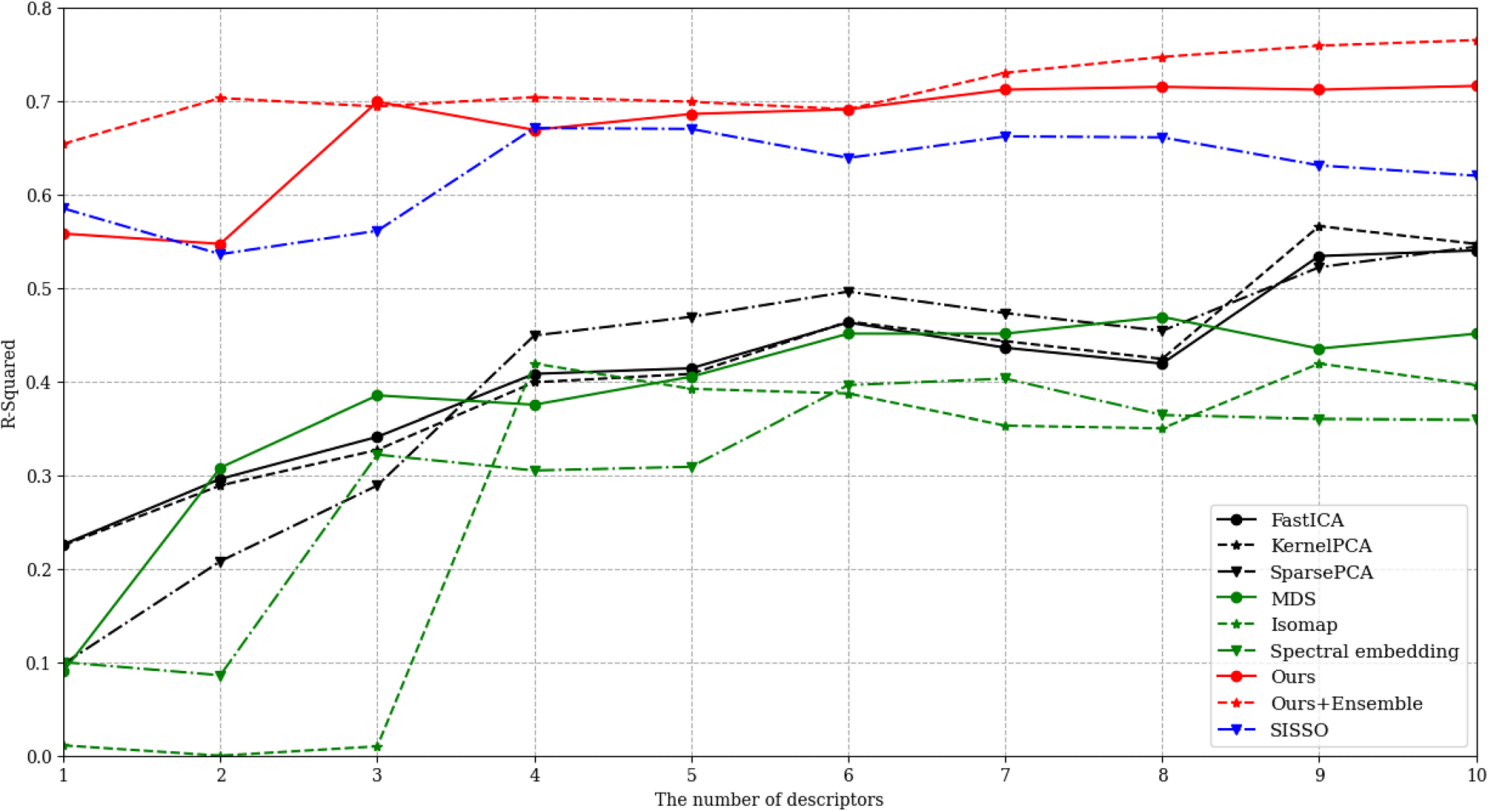
Comparison of R-squared values of ridge regression models for the proposed and comparison methods. Green and black lines are R-squared values of PCA and manifold learning based method, respectively. Red lines are R-squared values of method based on GDS.

In this section, we provide a quantitative analysis of which descriptors-that have either been selected from other algorithms such as machine learning feature extraction methods or generated by GDS for the $\Delta G_H$ prediction of TMDs-, can achieve higher predictive performance. We divided the dataset of 70 TMDs, 52 samples for training and 18 samples for test. We employed ridge regression to ensure reliable convergence across all methods. We progressively increased the number of descriptors from 1 to 10 and compared their performance for each method. For a fair comparison, we repeated the experiment five times, each time using different splits of the dataset. The results obtained from these iterations were averaged to provide a representative measure of performance. The comprehensive results are reported in Fig. 3 and detailed information of results are reported in Tables SI1 and SI2.

We used six feature extraction methods along with SISSO^23^ for comparison. The six feature extraction methods comprising three principal component analysis (PCA) methods: FastICA^37^, SparsePCA^38^, and KernelPCA^39^, and three manifold learning method: multi-dimensional scaling^40^(MDS), Isomap^41^, and spectral embedding^42^. These feature extraction methods are widely used in the field of machine learning^43,44^.

As depicted in Fig. 3, the addition of descriptors generally improves the predictive performance for all models. Nonetheless, the prediction model with the proposed method can be seen to outperform all of the comparison algorithms, regardless of the number of descriptors used. Moreover, it is worth emphasizing that while SISSO exhibits initial performance improvements, it eventually reaches a point where further enhancements are not observed. In contrast, our algorithm consistently demonstrates a continuous improvement over the number of descriptors, maintaining a steady and progressive enhancement. These results highlight that the proposed algorithm is capable of generating descriptors that are substantially related to $\Delta G_H$ from primary features.

Furthermore, some comparison methods tend to represent unstable performance with large variations when a large number of descriptors are included in the prediction model (typically six or more) due to noise or overfitting. On the other hand, in the case of the prediction model using the proposed method, it performed sufficiently well with only a few descriptors and showed stable performance with an increasing number of descriptors. This property can be attributed to the inherent randomness in the descriptor search process of the proposed algorithm, which allows for various aspects of the data to be examined. The genetic algorithm at the core of the proposed algorithm generates various descriptors, even with the same selected primary features, thus making it suitable for constructing ensemble^45^ models, the result of which is also shown in Fig. 3. Averaging three regression models learned with different descriptor sets, the ensemble model highlights the benefits of the inherent randomness in the proposed algorithm. Moreoever, as the number of descriptors increased, the variance of the performance based on GDS decreased, while the variance of other algorithms increased, as can be seen in the table in the supplementary material. This result can be considered to represent an improvement in the regression model’s robustness, which can be attributed to the complementary descriptors generated by the proposed algorithm.

### Qualitative analysis based on t-SNE visualization

**Figure 4:**
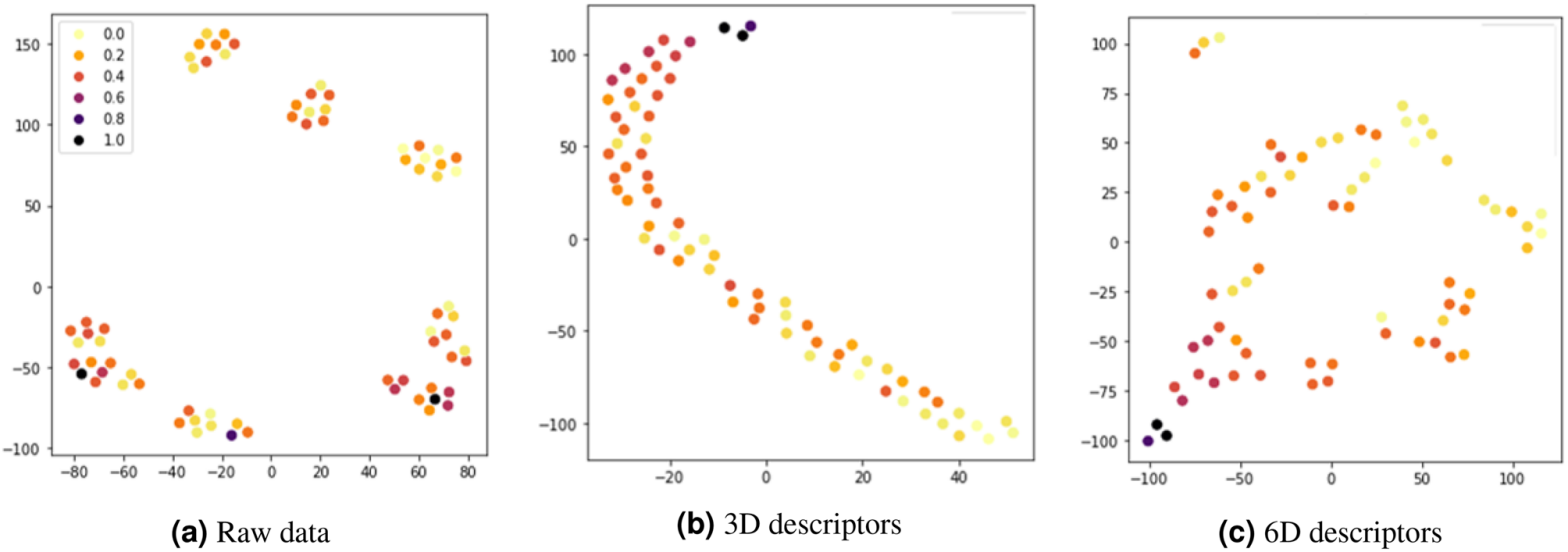
t-SNE visualization of raw data and descriptors. (**a**) is directly from the raw data, and plots (**b**, **c**) are from the descriptors we found.

To verify how the descriptors generated by the proposed algorithm were related to the target, $\Delta G_H$, we compared the primary features of 2D-TMDs and the descriptors by visualizing them using the t-SNE method. The visualization result is shown in Fig. 4, where the x-axis and y-axis of each figure are arbitrary axes without any inherent meaning or unit, and the position of the data point on the 2-dimensional space of each figure is determined by the similarity between data points on the feature space. In addition, the scaled $\Delta G_H$ of each data point is expressed in color.

In Fig. 4a, no relationship can be found between the distributions of the primary features and $\Delta G_H$; the raw primary features have little direct relationship with the $\Delta G_H$. The visualization results for the generated descriptors are shown in 4b and 4c. In 4b, it can be seen that a small number (3) of descriptors make the samples form one cluster and are simultaneously aligned in accordance with $\Delta G_H$, while 4c shows that the number and shape of clusters change when the descriptors are added. Nevertheless, the samples are aligned with the $\Delta G_H$ in the same way in both situations. Moreover, as shown in 4c, data of high $\Delta G_H$ and low $\Delta G_H$ can be more clearly separated when the number of descriptors is large. Through these results, we found that the proposed algorithm successfully generates the descriptors with a meaningful relationship with the target, $\Delta G_H$, by using the primary features. Although Fig. 4b shows that only a small number of descriptors can also have a clear relationship with the target variable, adding more descriptors to the predictor can result in performance improvement as shown in Fig. 3.

### Analysis of generated descriptors

**Table 1:** 10 descriptors obtained from each dimension level and the coefficients of ridge regression model.

Dimension	Trial 1	coefficient	Trial 2	coefficient
1	$\frac{Rx}{Rtm-Vtmx^2+DVEtm \times Vtmx}$	−0.190	$\frac{Qtm}{Vtmx^2-DVEtm \times Vtmx -Rx}$	0.173
2	$\frac{ |Ntmf|}{Vtm-WFx}$	0.075	$\frac{BEtmx \times Qx}{Vtm \times Wtm}$	0.320
3	$\frac{Rx}{Vtm \times Etm \times Wtm}$	−0.042	$\frac{Rx}{Vtm^2 \times Wtm}$	0.001
4	$\frac{Rx}{Vtm^2 \times Wtm}$	−0.012	$\frac{Rx\times BEtmx}{Wtm \times Vtm}$	0.017
5	$tan\left( \frac{BEtmx}{Wtm \times Vtm^2}\right)$	−0.002	$\frac{Qtm}{DVEtm -WFtm}$	0.000
6	$\frac{Qx^2}{WFtm\times Vtm^2}$	0.025	$\frac{Rx}{(Ctm-Nxf)\times (Qx-Vtm)}$	0.069
7	$\frac{Rx}{Vtm^2 \times Wtm}$	−0.079	$\frac{Vtm-DVEx}{Wtm-Qx}$	−0.001
8	$\frac{Covh}{(Ctm-Qx)\times (Cx-Vtmx)}$	0.000	$\frac{-0.108}{Qtm-Vtmx}$	0.042
9	$\frac{Vtm \times Wtm}{Qx}$	0.108	$cos(LEf + Rtm)$	0.002
10	$log\left( \frac{Rx}{Vtm \times (Ntm+Wtm)}\right)$	−0.059	$\frac{Etm}{Qtm-WFtm}$	0.052

We analyzed the effectiveness of descriptors generated by the proposed genetic algorithm by utilizing relevant literature in the field. As genetic algorithm utilized in GDS has inherent randomness, there may be variances in the descriptor search results across multiple trials. To account for the variability, we sampled two sets of descriptors for analysis. The results of 10*D* descriptors obtained through GDS and the coefficient values of ridge regression model are presented in Table 1. By examining the regression coefficients in the 1, it can be inferred that there is no evidence of overfitting in the model. The results of descriptor search through comparative methods are presented in Table SI4 in supplementary materials and unreported results are the results of methods for generating descriptors that are not explicitly expressed through mathematical formulas.

In both trials, the proposed method used Rx, Vtmx, and DVEtm to create the 1*D* descriptors. In one trial, Rtm was included as an additional feature in the descriptors, while the other trial used Qtm instead.

Rx represents the radius of the covalent bond of the chalcogen element. Through various experiments and theoretical studies, it has been proved that the $\Delta G_H$ can be controlled by changing the chalcogen atom in TMD materials^7,46–52^. It has been reported that MS$_2$ exhibits stronger hydrogen adsorption than MSe$_2$ and MTe$_2$, (M = transition metals), because S has a much shorter covalent radius compared to Se and Te^7^.

Vtmx refers to the average valence electron number of TM-X bond(where TM is the transition metal and X is the chalcogenide element in TMD materials). Studies by Liu et al.^53^ reported the hydrogen adsorption mechanism due to the interaction of the chalcogen element with the outermost valence electron of TM. Li et al.^54^ reported experimental results of controlling the hydrogen adsorption energy in a wide range by adjusting the electron density at the adsorption site by changing the average number of valence electrons in TM and X.

DVEtm is the distance to the outermost electron of the transition metal element, while Rtm is the covalent radius of the transition metal element. When TM-X forms a bond in a TMD material, it forms in two phases: trigonal prism (H phase) and octahedral prism (T phase), which is determined by the radius ratio of TM and X of the TM-X bond^55^. In the TMD system, the $\Delta G_H$ substantially changes according to the phase change, and the 1T structure is known to have a very strong hydrogen adsorption strength^56–58^. In general, the chemical bonds between atoms in a TMD material are known to have a nature that is both ionic and covalent^59,60^, and the ionic characteristics are interpreted as described by DVEtm, while the covalent characteristics are interpreted as described by Rtm.

Qtm is the principal quantum number of the transition metal. The transition metals mainly used in the TMD system are divided into three periods with 3d, 4d, and 5d orbitals. Many studies have been conducted in attempts to control $\Delta G_H$ of TMD materials by varying the principal quantum number in one group of transition metals^61–67^. Chia et al. calculated the MX2 system (M = V, Nb, and Ta) and reported that the $\Delta G_H$ was significantly changed by the change of the principal quantum number^67^.

In addition to the features described above, Vtm and Wtm are repeatedly included for the entire descriptors. Vtm means the number of valence electrons of a transition metal element. In the periodic table, transition metals are divided into groups according to the number of electrons in their valence shell. Groups 4, 5, and 6 are called early transition metals, and groups 7–12 are typically classified as late transition metals. In Lee et al.^68^’s study, early TM MX$_2$ (M = group 4–6) is a metallic system, whereas late TM MX$_2$ (M = group 7–12) is a semiconducting system, so hydrogen adsorption is preferred in the early TM MX$_2$ system^68^. Several research groups have also reported that the difference in the number of electrons in the outermost shell greatly affects $\Delta G_H$^68,69^.

Wtm is the atomic weight of transition metal. Since the atomic weight is set as a unique value for each element, it corresponds to a unique feature, like a fingerprint, of a transition metal, unlike other primary features. That is, the change in Wtm is correlated with the change in all physical properties of the transition metal, including the atomic radius, principal quantum number, and valence electron number described above. Although the atomic radius, principal quantum number, and valence electron count typically appear as overlapping values in several TMs, the atomic weight is significantly different for each TM. Therefore, Wtm can be an important primary feature when describing $\Delta G_H$ by the change in transition metal atoms.

## Conclusion

In this paper we present Genetic Descriptor Search (GDS), a new descriptor search algorithm that predicts TMD’s property using self-exploring scheme through a genetic algorithm. GDS first create an initial population of trees and evolve them iteratively using the genetic process. Then, GDS select the tree that best describes the target property as the descriptor. This process is repeated until the desired number of descriptors is obtained. Our experimental results demonstrated both the effectiveness and explainability of the proposed method on the property prediction task. We also verified that the obtained descriptors contain variables that are consistent with chemical knowledge. Consequently, the proposed method, GDS, is a highly effective approach that improves both the performance and computational efficiency of existing descriptor search algorithms. In fields of the development of new 2D materials, GDS enables efficient screening and accurate prediction of material properties and provides valuable insights into the relationship between material structure and properties. In our case, we analyzed the relationship between the primary features of 2D TMD materials and the hydrogen adsorption free energy, and the ridge regression model with GDS selected MnS_2_ with chalcogen vacancy, FeS_2_ with chalcogen vacancy, and TaS_2_ with chalcogen vacancy as the best materials for catalytic performance. Detailed the hydrogen adsorption free energy prediction results are reported in Table SI5 in Supplementary materials. The proposed algorithm, GDS, can also leverage the domain expertise in several ways such as restricting the primary feature and operations included in the same subtree. Thus, the performance of GDS can further be improved by utilizing the domain knowledge in future studies. In addition, since the GDS is not task-specific, it can be applied to the diverse tasks and chemical properties.

### Supplementary Information

Supplementary Information.

## Data Availability

The codes for the proposed method and dataset used in this study are readily accessible at https://github.com/andrew0411/BRL_project2_GDS/tree/main.
