# Supplementary material for: Genetic descriptor search algorithm for predicting hydrogen adsorption free energy of 2D material
Source: Sci Rep. 2023 Aug 5;13:12729. doi: 10.1038/s41598-023-39696-0 (PMC10404247; doi:10.1038/s41598-023-39696-0)
Supplement: Supplementary file 1 — Supplementary Information. [file 41598_2023_39696_MOESM1_ESM.pdf]

Supplementary Information on Genetic descriptor search algorithm for predicting hydrogen adsorption free energy of 2D material

Jaehwan Lee[1,2,+], Seokwon Shin[1,2,+], Jaeho Lee[3], Young-Kyu Han[3,*], Woojin Lee[4,*], and Youngdoo Son[1,2,*]

[1]Department of Industrial and Systems Engineering, Dongguk University – Seoul, Seoul 04620, South Korea
[2]Data Science Laboratory (DSLAB), Dongguk University – Seoul, Seoul 04620, South Korea
[3]Department of Energy and Materials Engineering, Dongguk University-Seoul, Seoul 04620, South Korea
[4]School of AI Convergence, Dongguk University – Seoul, Seoul 04620, South Korea
[+]These authors are contributed equally to this work.
[*]Corresponding authors: ykenergy@dongguk.edu; wj926@dgu.ac.kr; youngdoo@dongguk.edu

A. Quantitative Evaluation Results

Table SI1. Average of R-squared values

	the number of descriptors									
Method	1	2	3	4	5	6	7	8	9	10
FastICA	0.226	0.296	0.341	0.408	0.414	0.463	0.436	0.419	0.534	0.540
KernelPCA	0.225	0.289	0.327	0.399	0.408	0.464	0.443	0.424	0.566	0.547
SparasePCA	0.098	0.208	0.289	0.449	0.469	0.496	0.473	0.454	0.522	0.544
MDS	0.090	0.308	0.385	0.375	0.405	0.451	0.451	0.469	0.435	0.451
Isomap	0.011	0.000	0.010	0.419	0.392	0.387	0.353	0.350	0.419	0.396
Spectral embedding	0.100	0.086	0.322	0.305	0.309	0.396	0.403	0.364	0.360	0.359
SISSO	0.585	0.536	0.561	0.671	0.670	0.639	0.662	0.661	0.631	0.620
**Ours**	0.558	0.547	0.699	0.669	0.686	0.691	0.712	0.715	0.712	0.716
**Ours + Ensemble**	0.654	0.703	0.694	0.704	0.699	0.691	0.730	0.747	0.759	0.765

Table SI2. Standard deviation of R-squared values

	the number of descriptors									
Method	1	2	3	4	5	6	7	8	9	10
FastICA	0.051	0.055	0.064	0.062	0.068	0.081	0.108	0.097	0.083	0.078
KernelPCA	0.052	0.054	0.065	0.061	0.068	0.082	0.112	0.129	0.104	0.112
SparsePCA	0.039	0.058	0.067	0.068	0.076	0.065	0.085	0.103	0.072	0.072
MDS	0.170	0.047	0.077	0.070	0.064	0.091	0.082	0.072	0.092	0.154
Isomap	0.074	0.081	0.084	0.088	0.100	0.111	0.203	0.215	0.147	0.150
Spectral embedding	0.070	0.083	0.081	0.085	0.094	0.094	0.113	0.166	0.166	0.168
SISSO	0.105	0.038	0.053	0.077	0.106	0.127	0.117	0.117	0.043	0.070
**Ours**	0.105	0.125	0.048	0.062	0.061	0.051	0.045	0.041	0.047	0.054

B. Primary Features of TMD Materials

Table SI3. All the 27 primary features used are listed, observable physical descriptors including anion, cationic, and local area that may affect catalytic performance

Feature	Description
Rtm	Radii covalent of transition metal atom (Å)
Etm	Electronegativity of transition metal atom (Pauling)[1]
Wtm	Atomic weight of transition metal atom $(10^{-3}kg)$
Qtm	Quantum number of transition metal
Ctm	Coordination number of transition metal
Vtm	Valence electrons number of transition metal
EItm	First ionization energy[2] of transition metal (KJ/mol)
DVEtm	Distance valence electron of transition metal (Schubert)[3] (A)
WFtm	Work function of transition metal (eV)
Ntm	Total number of transition metals
Ntmf	The number of transition metal atoms nearest to the adsorption site
Nx	Total number of chalcogenide atoms
Vx	Valence electrons number of chalcogenide atoms
EIx	Energy ionization first of chalcongenide atoms (KJ/mol)
Nxs	The number of chalcogenide atoms next-nearest to the adsorption site
Nxf	The number of chalcogenide atoms nearest to the adsorption site
Cx	Coordination number of chalcogenide atoms
Qx	Quantum number of chalcogenide atoms
Wx	Atomic weigh of chalcogenide atomst $(10^{-3}kg)$
WFx	Work function of chalcogenide atoms (eV)
Rx	Radii covalen of chalcogenide atoms (Å)
DVEx	Distance valence electron of chalcogenide atoms (Schubert) (A)
LEs	Nearest neighbor local electronegativity (Etm * Ntmf + Nx *Nxf)
LEf	Next-nearest neighbor local electronegativity (Nx * Nxs)
Vtmx	Average valence electron number of TM-X (Vtm / Ctm + Vx / Cx)
BEtmx	Bond electronegativity of TM-X[4,5]
Covh	Hydrogen coverage[6]

C. Implementation Details of SISSO algorithm

We evaluated the performance of SISSO using the source codes provided by the authors of the original paper[7] with our dataset. We controlled the number of descriptors included in the final predictor from one to ten for fair comparison. Any truncation based on the estimation error was not applied. In addition, we limited the number of primary features included in the descriptor candidates for each SIS process to three, which ensures fair comparison among the proposed and benchmark methods without significant discrepancy in computational cost.

References

1. Pauling, L. Citation classic-the nature of the chemical-bond and the structure of molecules and crystals-an introduction to modern structural chemistry. *CURRENT CONTENTS/PHYSICAL CHEMICAL & EARTH SCIENCES* 16–16 (1985).
2. Moore, C. E. Ionization potentials and ionization limits derived from the analyses of optical spectra. Tech. Rep., NATIONAL STANDARD REFERENCE DATA SYSTEM (1970).
3. Xu, Y., Yamazaki, M. & Villars, P. Inorganic materials database for exploring the nature of material. *Jpn. J. Appl. Phys.* **50**, 11RH02 (2011).
4. Li, K., Wang, X., Zhang, F. & Xue, D. Electronegativity identification of novel superhard materials. *Phys. Rev. Lett.* **100**, 235504 (2008).
5. Ran, N. *et al.* Bond electronegativity as hydrogen evolution reaction catalyst descriptor for transition metal (tm= mo, w) dichalcogenides. *Chem. Mater.* **32**, 1224–1234 (2020).

Table SI4. Descriptor search results using the comparison methods

Dimension	SparsePCA	FastICA	SISSO
1	$0.5Elx - 0.4Qx - 0.4Wx + 0.5WFx - 0.5Rx$	$0.7Rtm + 0.6Etm + 0.6Vtm + 2.5DVEtm + 0.8WFtm$	$\|WFx + Vtmx\|$
2	$0.6Ntm + 0.1Ntmf - 0.6Cx + 0.2Vtmx + 0.4Covh$	$-3.1Wtm + 2.4Qtm + 0.6Vtm - 0.8DVEtm$	$DVEtm - WFtm$
3	$-0.6Ctm - 0.6Nx + 0.4Covh$	$0.7Ctm + 0.7Nx - 0.9Covh$	$Qx * Nxs$
4	$0.3Etm + 0.7Vtm + 0.5Eltm + 0.4WFtm + 0.2Vtmx$	$0.6Rtm - 1.4Wtm - 0.7Qtm - 2.0Eltm + 2.2DVEtm + WFx + 1.1DVEx$	$Ctm + Cx$
5	$0.5Rtm + 0.9Qtm$	$0.5Wtm + 0.8DVEtm - 0.9Nxf - 0.6LEf$	$\|Wtm - Wx\|$
6	$-0.1Ntmf - 0.8Nxf - 0.5LEf$	$1.5Rtm - Wtm + 1.2Qtm - Eltm + 1.1DVEtm + Nxf - 0.7WFx - 0.9DVEx - 0.5BEtmx$	$Rtm + BEtmx$
7	$-0.3Ntmf - 0.8Nxs - 0.5LEs - 0.1BEtmx$	$-0.5Rtm - 0.6Wtm - 0.9DVEtm + 1.6WFx + 3.2DVEx + 0.8BEtmx$	$\|Nxf - Qx\|$
8	$DVEtm$	$-0.9Ntm - 0.9Ntmf - 1.5Nxs - 0.8Nxf - 0.9Cx - 0.7LEf - LEs + 0.5Covh$	$\|Qtm - Ntmf\|$
9	Wtm	$-1.5Qtm - 0.6Elx + 0.9Nxs - 2.2WFx + 0.6Rx - 2.5 * DVEx - 1.1BEtmx$	$\|Nxs - Nxf\|$
10	$-0.1Qx - DVEx$	$-0.9Nxs + 0.8Nxf - 0.6LEs$	$\|Vtm/Nxf\|$

Table SI5. Prediction results with descriptors identified by GDS for the 4 top with ΔG_H closest to zero

Materials	Predicted ΔG_H (eV)
MnS2 with chalcogen vacancy	0.00
TaS2 with chalcogen vacancy	0.03
FeS2 with chalcogen vacancy	-0.01
VSe2 with chalcogen vacancy	0.04

6. Tsai, C., Chan, K., Nørskov, J. K. & Abild-Pedersen, F. Theoretical insights into the hydrogen evolution activity of layered transition metal dichalcogenides. *Surf. Sci.* **640**, 133–140 (2015).

7. analytics-compressed-sensing (2022). https://gitlab.mpcdf.mpg.de/nomad-lab/analytics-compressed-sensing.
